# Structural mechanism of tapasin-mediated MHC-I peptide loading in antigen presentation

**DOI:** 10.1038/s41467-022-33153-8

**Published:** 2022-09-17

**Authors:** Jiansheng Jiang, Daniel K. Taylor, Ellen J. Kim, Lisa F. Boyd, Javeed Ahmad, Michael G. Mage, Hau V. Truong, Claire H. Woodward, Nikolaos G. Sgourakis, Peter Cresswell, David H. Margulies, Kannan Natarajan

**Affiliations:** 1 Molecular Biology Section, Laboratory of Immune System Biology, National Institute of Allergy and Infectious Diseases, National Institutes of Health, Bethesda, MD 20892-1892 USA; 2 Department of Biochemistry and Biophysics, Perelman School of Medicine, University of Pennsylvania, Philadelphia, PA 19104 USA; 3 Center for Computational and Genomic Medicine and Department of Pathology and Laboratory Medicine, Children’s Hospital of Philadelphia, Philadelphia, PA 19104 USA; 4 Department of Immunobiology, Yale University School of Medicine, New Haven, CT 06520-8011 USA; 5 Present Address: Department of Biological Engineering, Massachusetts Institute of Technology, Cambridge, MA 02139 USA

**Keywords:** Antigen processing and presentation, X-ray crystallography, Peptides, Chaperones

## Abstract

Loading of MHC-I molecules with peptide by the catalytic chaperone tapasin in the peptide loading complex plays a critical role in antigen presentation and immune recognition. Mechanistic insight has been hampered by the lack of detailed structural information concerning tapasin–MHC-I. We present here crystal structures of human tapasin complexed with the MHC-I molecule HLA-B*44:05, and with each of two anti-tapasin antibodies. The tapasin-stabilized peptide-receptive state of HLA-B*44:05 is characterized by distortion of the peptide binding groove and destabilization of the β_2_-microglobulin interaction, leading to release of peptide. Movements of the membrane proximal Ig-like domains of tapasin, HLA-B*44:05, and β_2_-microglobulin accompany the transition to a peptide-receptive state. Together this ensemble of crystal structures provides insights into a distinct mechanism of tapasin-mediated peptide exchange.

## Abstract (web summary)

The catalytic chaperone tapasin assists peptide loading onto MHC-I molecules for antigen presentation and immune recognition. Here, the authors present crystal structures that provide insights into the molecular mechanism of tapasin-mediated peptide exchange.

## Introduction

Cell surface display of peptide–Major Histocompatibility Complex class I (pMHC-I) molecules plays a critical role in T cell and natural killer (NK) cell immunity^1,2^. The intracellular loading of peptides onto MHC-I is largely dependent on the activity of the PLC^3^, a multimolecular complex consisting of the transporter associated with antigen processing (TAP)1/2, tapasin, calreticulin, ERp57, and the MHC-I–β_2_m heterodimer. Tapasin, a key component of the PLC, is a 48 kDa transmembrane glycoprotein that bridges the TAP transporter and MHC-I to facilitate peptide loading^4–8^. Cell lines and mice lacking tapasin express reduced levels of surface MHC-I^6,9^ affecting antigen presentation, CD8^+^ T cell development, and anti-viral immunity^10,11^. Tapasin’s importance in antigen presentation is underscored by its role in human disease, exemplified by bare lymphocyte syndrome type 1, caused by deletion of the tapasin-encoding *TAPBP* gene^12^. Many tumors escape immune surveillance by targeting components of the PLC, including tapasin^13–16^. Tapasin’s influence on expression of human HLA-A, -B, and -C molecules impacts the outcomes of viral infection^17–19^. Despite an X-ray structure of a tapasin–ERp57 complex^20^, molecular dynamics (MD) studies of the PLC^21,22^, NMR studies of the tapasin–MHC-I interaction^23^, and cryo-EM visualization of the PLC^24^, the mechanism by which tapasin exercises its peptide loading function remains to be elucidated. The determination of structures of the tapasin-MHC-I complex at high resolution either by crystallography or cryo-EM has posed serious challenges in past decades, due in part to the dynamic flexibility of tapasin and MHC-I domains. Recent functional, X-ray, and NMR studies of the tapasin homolog, TAPBPR, provide some insight into details of tapasin function^25–30^. However, although TAPBPR reveals similarity in function to tapasin, it is not a component of the PLC. Thus, differences between the expression and function of TAPBPR and tapasin and allelic differences among MHC-I molecules with respect to tapasin dependence^19,31,32^ demand further exploration of the tapasin–MHC-I interaction. Specific unresolved details include the function of several loops of tapasin inferred from studies of TAPBPR^27^.

Here, we describe a crystal structure of a tapasin–HLA-B*44:05–β_2_m complex (determined at 3.1 Å resolution) as well as structures of tapasin complexed with Fab fragments of two well-characterized monoclonal antibodies. These structures, complemented by binding studies and NMR data, reveal details of tapasin function, emphasize the dynamic nature of the tapasin–MHC-I interaction, highlight differences and similarities to TAPBPR, and substantiate a revised mechanistic model.

## Results

### Interaction of tapasin with HLA-B*44:02 and HLA-B*44:05

**Fig. 1:**
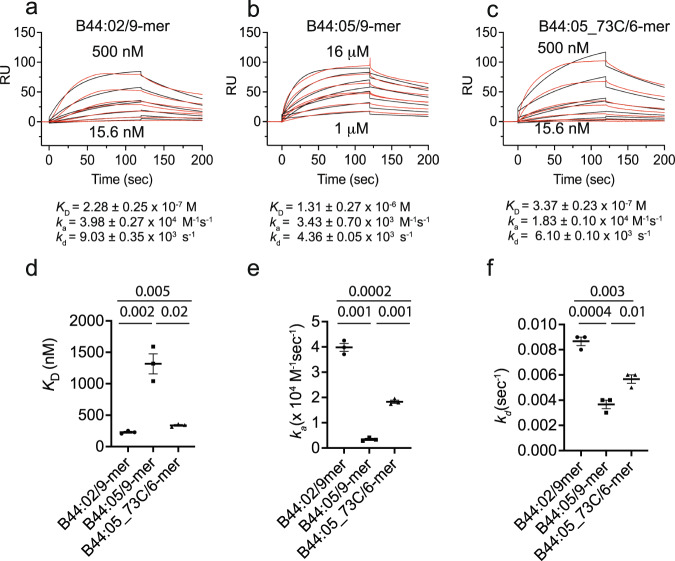
Affinity and kinetics of the interactions between tapasin and B44:02 and B44:05. **a** SPR analyses of the interaction of biotinylated tapasin captured on a streptavidin chip and B44:02–9mer. **b** B44:05–9mer. **c** B44:05-T73C–6mer. Data are shown in red and fits of the data to a 1:1 binding model are overlaid in black. **d** equilibrium constant *K*_*D*_, **e** association rate constant *k*_*a*_ and **f** dissociation rate constant *k*_*d*_. Kinetics data consisting of (*n* = 3) independent measurements of the same preparations of tapasin and MHC-I molecules of the indicated interactions are shown below each panel as mean and as scatter plots. p values, calculated by two-tailed unpaired *t*-test as implemented in GraphPad Prism, are indicated. These data are representative of similar measurements performed with multiple preparations over a span of two years. Source data are provided as a source data file.

HLA-B*44:02 and HLA-B*44:05 (B44:02 and B44:05 hereafter), represent tapasin-dependent and tapasin-independent MHC-I molecules, respectively, based on their cell surface expression in tapasin-deficient cell lines^19,31,32^. The two allelomorphs differ at a single residue in the F pocket region, Asp116 in B44:02 and Tyr116 in B44:05. When loaded with the same 9mer peptide (EEFGRAFSF), B44:02 consistently displayed an ~6-fold higher affinity for tapasin than did B44:05 (*K*_D_ = (2.28 ± 0.25) x 10^−7^ M versus (1.31 ± 0.27) x 10^−6^ M) (Fig. 1), attributable to a higher association rate constant. Since MD simulations^22,33,34^ and NMR data^23^ suggest that increased dynamics of the F pocket region of MHC-I favor tapasin binding^35^, we asked whether increasing F pocket flexibility of the weaker binding B44:05 allelomorph would improve its affinity for tapasin. Using an approach previously applied to the TAPBPR–MHC-I interaction^28^, we replaced Thr73 in the α1 helix of the B44:05 heavy chain with Cys and refolded it with human β_2_m and a C-terminally truncated 6mer variant (EEFGRC) of the original 9mer peptide. The F pocket is loosely conformed by inclusion of a GlyLeu dipeptide in the folding reaction^36^. This strategy permits greater dynamics of the C-terminal portion of the peptide binding groove potentially allowing enhanced interactions with tapasin. As shown in Fig. 1b, c the kinetics of the interaction of tapasin with B44:05-T73C–6mer revealed an ~4-fold higher affinity than with the parental B44:05–9mer. This result is consistent with the view that F pocket dynamics influence tapasin interaction. Efforts to produce B44:02-T73C–6mer molecules in good yield were unsuccessful.

### Structure of the Tapasin–B44:05-T73C–6mer complex

**Fig. 2:**
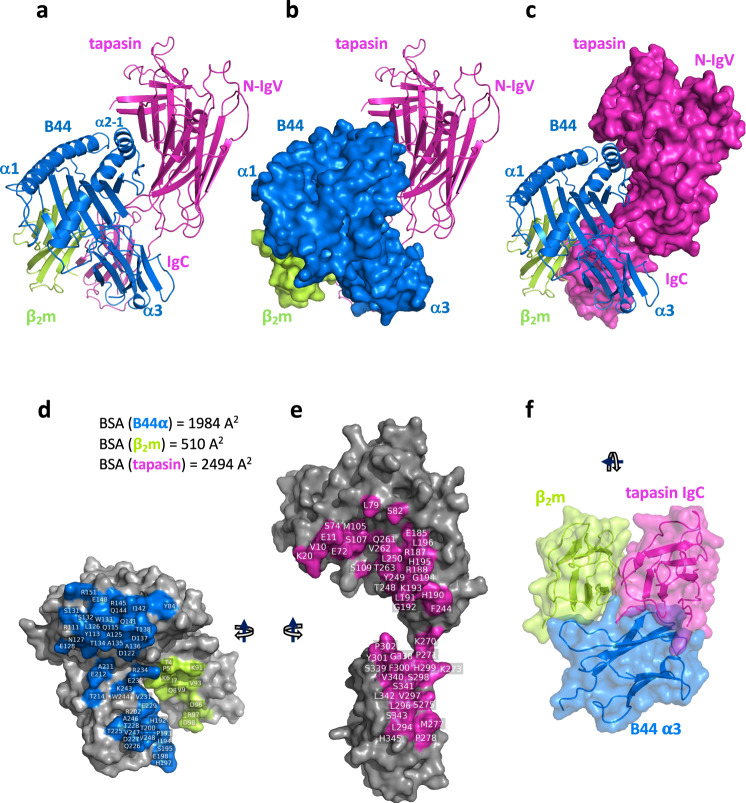
Overall structure of the Tapasin–B44:05–6mer complex. **a** Ribbon representation of Tapasin (magenta), B44:05 heavy (α) chain (blue), and β_2_m light chain (lime). **b** Similar to **a**, but with B44:05 α and β_2_m in surface display. **c** tapasin in surface display. **d** Interface residues of B44:05 α and β_2_m in surface display, with residues colored and numbered. **e** Interface residues of Tapasin as indicated. **f** Rotated view of the membrane proximal trimer of B44:05 α3 domain (blue), β_2_m (lime), and Tapasin IgC domain (magenta). BSA Buried surface area.

We obtained crystals of a complex of tapasin with B44:05-T73C–6mer (Table 1), and solved the structure by molecular replacement as described in Methods. The structure of the complex (Fig. 2) was refined to R_work_/R_free_ of 28.4/30.8% (Table 1). For comparison we determined the crystal structures of unliganded B44:05-T73C–6mer (Supplementary Fig. 1a) and B44:05-T73C–9mer (Supplementary Fig. 1b) as well as tapasin bound to two well-characterized antibody Fab fragments.

**Table 1:** X-ray data collection and refinement statistics

	B44:05-T73C–9mer	B44:05-T73C–6mer	Tapasin–B44:05-T73C–6mer	Tapasin–PaSta1 (Fab)	Tapasin–PaSta2 (Fab)	PaSta2 (Fab)
PDBID	7TUC	7TUD	7TUE	7TUF	7TUG	7TUH
**Data collection**						
Space group	P2_1_2_1_2_1_	P2_1_2_1_2_1_	P6_1_	P2_1_	C222_1_	P2_1_
**Cell dimensions**						
*a*, *b*, *c* (Å)	50.75, 82.58, 110.89	49.17, 79.76, 107.21	116.87, 116.87, 131.14	117.42, 68.99, 142.98	101.09, 168.82, 108.66	49.10, 62.13, 128.46
*α, β, γ* (°)	90.0, 90.0, 90.0	90.0, 90.0, 90.0	90.0, 90.0, 120.0	90.0, 108.42, 90.0	90.0, 90.0, 90.0	90.0, 90.04, 90.0
Resolution (Å)*	38.70-1.25 (1.30–1.25)	39.88-1.45 (1.50–1.45)	55.03-3.11 (3.22–3.11)	47.45-2.80 (2.69–2.80)	46.04-3.90 (4.04–3.90)	55.93-2.30 (2.38–2.30)
*R*_*sym*_ *or R*_*merge*_	0.033 (0.413)	0.071 (0.943)	0.337 (0.978)	0.087 (1.326)	0.107 (1.416)	0.109 (1.933)
*I/σ(I)*	11.6 (1.4)	11.6 (1.1)	3.2 (0.9)	10.4 (1.0)	7.5 (0.9)	8.6 (0.78)
Completeness (%)	95.4 (80.3)	99.0 (97.0)	99.8 (99.6)	97.7 (95.3)	95.2 (92.7)	99.0 (98.8)
Redundancy	2.0 (2.0)	4.8 (4.1)	5.8 (6.0)	3.8 (3.7)	3.5 (3.4)	5.8 (5.8)
*R*_*pim*_	0.033 (0.413)	0.036 (0.509)	0.149 (0.435)	0.052 (0.821)	0.065 (0.851)	0.049 (0.866)
CC_1/2_	0.996 (0.555)	0.998 (0.553)	0.940(0.587)	0.998 (0.483)	0.998 (0.477)	0.997 (0.669)
Estimated twin fraction	0.0 (none)	0.00 (none)	0.307 (h,-h-k,-l)	0.0 (none)	0.0 (none)	0.460 (h,-k,-l)
**Refinement**						
Resolution (Å)*	38.70-1.25 (1.30–1.25)	39.88-1.45 (1.50–1.45)	55.03-3.11 (3.22–3.11)	47.45–2.80 (2.69–2.80)	46.04–3.90 (4.04–3.90)	55.93–2.30 (2.38–2.30)
No. reflections^**§**^	123417 (1935)	74688 (3734)	18395 (920)	52731 (1998)	8234 (409)	34308 (1716)
*R*_work_/*R*_free_ (%)	18.0/19.5 (26.5/26.1)	19.2/21.3 (29.0/32.0)	28.4/30.8 (31.6/34.2)	23.7/27.6 (38.0/42.9)	28.2/33.2 (35.1/38.3)	21.8/24.0 (29.6/33.0)
No. atoms	3650	3752	5630	11290	5304	6606
Protein	3172	3155	5630	11209	5290	6466
Water + ligands	454 + 24	583 + 14	0	53 + 28	14	140
B-factor Wilson/Ave	9.5/15.5	18.3/24.5	80.3/71.8	51.2/65.4	67.6/78.2	41.3/41.7
Protein	14.2	22.8	71.8	67.9	78.2	41.8
Water + ligands	24.1 + 25.3	33.3 + 38.6	0	40.6 + 67.1	89.9	37.8
**R.m.s. deviations**						
bond length (Å)	0.004	0.009	0.002	0.003	0.002	0.002
bond angle (°)	0.81	0.93	0.66	0.66	0.52	0.54
**Ramachandran**						
favored (%)	98.9	98.9	86.2	90.37	89.9	90.9
allowed (%)	1.1	1.1	11.4	6.7	8.0	7.8
outliers (%)	0.0	0.0	2.4	2.9	2.0	1.3
**Molprobity**						
clashscore	1.92	7.29	12.7	8.8	3.75	7.03

(*Values in parenthesis are for highest resolution shell, ^§^Values in parenthesis are the number of reflections for *R*_free_).

Tapasin binds to a broad surface of the external aspect of B44:05 including the α2-1 helix, the α3 domain, and β_2_m (Fig. 2a–c). The interface buries an area of 2494 Å^2^, of which 1984 Å^2^ is contributed by the MHC-I heavy chain and 510 Å^2^ by β_2_m (Fig. 2d, e, Supplementary Table 1 for list of contacts). The contacts of tapasin to B44:05 are mediated by loops that bind the MHC-I α1α2 platform domain and those that interact with the membrane proximal α3 and β_2_m domains. The tapasin N–IgV domain forms a glove-like surface that addresses the MHC-I platform and cradles the α2-1 helix and supporting β7 and β8 strands and connecting loops (Fig. 2a and Supplementary Fig. 2). Superposition of the N–IgV domain of tapasin–B44:05 on the same domain of tapasin–ERp57 (PDB 3F8U) reveals movement of the IgC domain (Supplementary Fig. 2c).

**Fig. 3:**
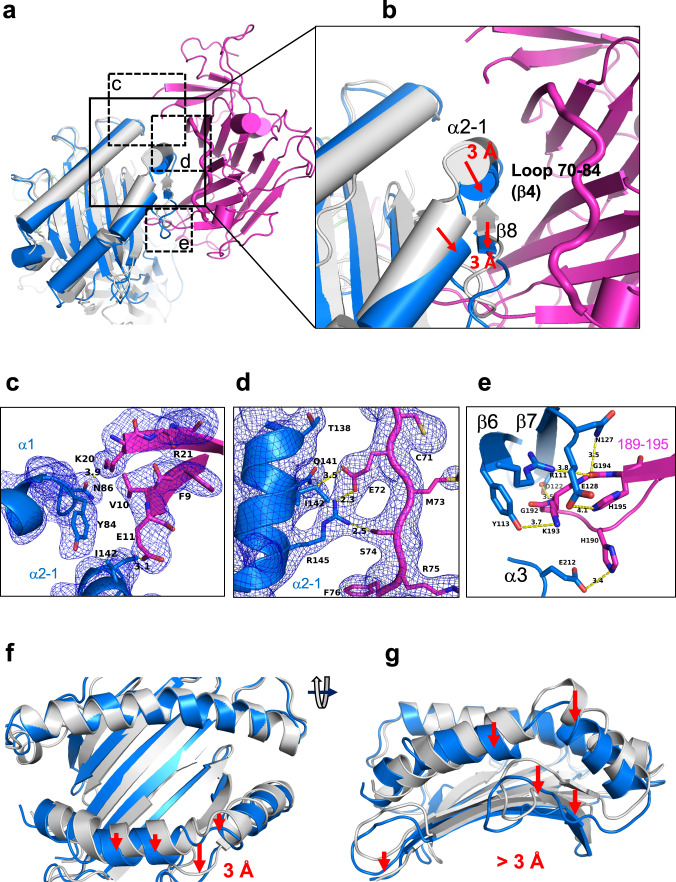
Comparison of unliganded B44:05-T73C-6mer (7TUD) with the complex of Tapasin–B44:05 (7TUE) reveals displacement of α2-1 helix and β-sheet floor of peptide binding groove of B44:05. The indicated models were superposed based on residues 54-84 of the MHC-I α chain. **a**–**d** Views of the tapasin–B44:05 interface are shown, with 1σ 2mFo-DFc map for **c**, **d**. Letters in panel **a** denote the regions enlarged in **c**, **d** and **e**. Movement of α2-1 helix (**a**, **b**, **f**, **g**), and **c**, local contacts of Glu11 and Lys20, and **d**, portion (Glu72-Phe76) of the long strand–loop Glu72-Lys84, and **e**, of the hairpin loop Gln189-His195 are shown. Yellow dotted lines indicate H-bonds with distances noted in Å.

Several tapasin loops interact with the MHC-I platform: that bounded by residues Glu11 and Lys20, of which amino acids 12 to 19 are not visualized in electron density; a long strand/loop that extends from residues Glu72 to Lys84; and the hairpin loop from Gln189 to His195 (Fig. 3a–e). Superposition of the platform domain of bound and free B44:05-T73C–6mer reveals deformations of the groove of the tapasin-bound state that are incompatible with peptide binding (Fig. 3f, g). Specifically, the α2-1 helix is drawn away from the groove by about 3 Å, and the floor of the groove is displaced downward by 3 Å (Fig. 3a, b, f, g), leaving the peptide binding groove of the tapasin–B44:05-T73C–6mer complex without electron density representing peptide or the GlyLeu dipeptide (Supplementary Fig. 3b) employed in the refolding of B44:05-T73C–6mer. Electron density for peptide and dipeptide was clearly discerned in the unliganded B44:05-T73C–6mer (Supplementary Fig. 3a).

For the Glu11-Lys20 loop in tapasin–B44:05, where no intervening electron density is observed (but which is clearly visualized in tapasin complexed to the Fab of PaSta1 (Supplementary Fig. 3c), Glu11 interacts with Ile142 of the α2-1 helix of B44:05 (Fig. 3c). Lys20 is 3.9 Å from the Oδ1 of the conserved Asn86 of B44:05, but still permits space for the N-linked glycan common to all MHC-I molecules (Supplementary Fig. 3d). The location of Glu11 and Lys20 precludes interaction of the connecting loop with the floor of the peptide-binding groove at the F pocket ruling out a scoop-like mechanism for displacing bound peptide^37^. Additionally, the positions of B44:05 residues Thr80, Lys146, Tyr84, and Ile142 in the tapasin–B44:05 complex (see figs.) prevent access of the loop to the F pocket. Moreover, the distance from the side chain of either Glu11 or Lys20 to Ile95 on the floor of B44:05 is at least 16 Å. The structure is consistent with a hovering peptide trap model based on NMR studies on the HLA-A*02–TAPBPR complex^30^. Additionally, Tyr84 of B44:05 directs its side chain towards the peptide binding groove, blocking access of the tapasin Glu11-Lys20 loop (Fig. 3c). The second B44:05-binding loop of tapasin, Glu72 to Lys84, a structural feature unique to tapasin and absent in TAPBPR, interacts with the α2-1 helix (including side chains Gln141, Arg145, and Glu148) of B44:05 (Fig. 3b, d). Residues Glu72 and Ser74 approach the α2-1 helix residues Ile142 and Arg145 (Fig. 3d), contributing to the displacement of the α2-1 helix. The third major tapasin loop, Gln189 to His195, protrudes beneath the β-sheet floor of the peptide binding groove to contact platform strands β6 and β7, also directing His190 towards Glu212 of the B44:05 α3 domain (Fig. 3e). In addition to these major interactions involving tapasin’s N–IgV domain, the IgC domain engages the membrane proximal domains of MHC-I through contacts with 16 residues of the α3 domain and 8 of β_2_m (Fig. 2d, f, Supplementary Table 1). This extensive interaction with β_2_m is of particular interest as it may weaken the β_2_m-dependent scaffolding of the peptide binding domain floor and prevent the well-documented cooperativity between peptide and β_2_m binding^38–40^. Consistent with this view, electron density corresponding to the 6mer peptide is lacking in the B44:05 groove in the tapasin complex (Supplementary Fig. 3b). The tapasin–B44:05-T73C–6mer complex provides a structural explanation for the behavior of mutants of both MHC-I^41–43^ and tapasin^20^ deleterious to the tapasin–MHC-I interaction (Supplementary Table 2).

### Structures of tapasin complexed with Fabs of PaSta1 and PaSta2

**Fig. 4:**
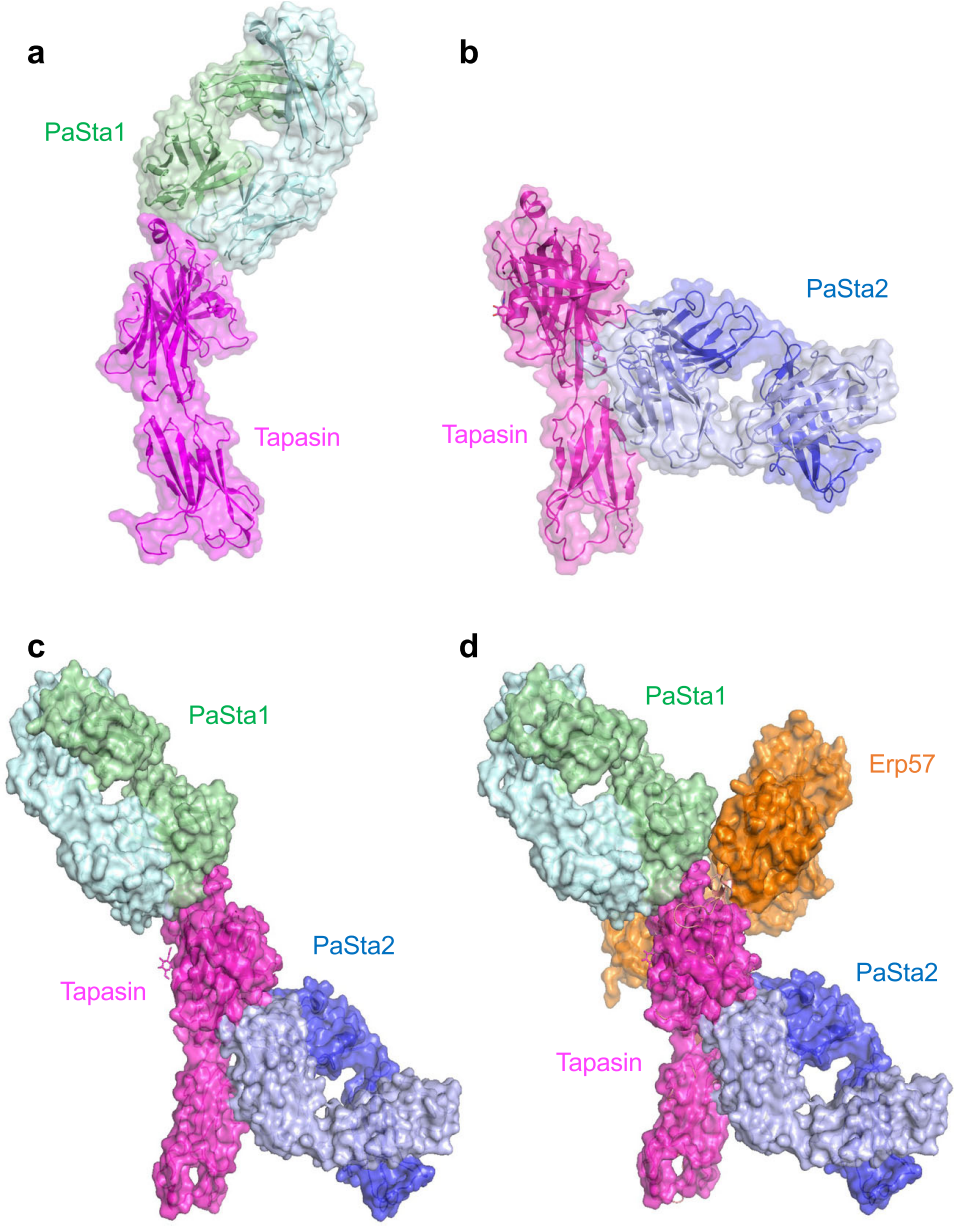
Structures of tapasin–PaSta1 (7TUF) and tapasin–PaSta2 (7TUG) complexes locate sites of antibody interactions. **a** Ribbon/transparent surface representation of the structures of the tapasin–PaSta1 complex. **b** tapasin–PaSta2 complex. **c** surface representation based on superposition of tapasin from each complex. **d** superposition of the PaSta1, PaSta2, and ERp57 (3F8U) complexes. For panel **a**, the first complex in the asymmetric unit is shown.

To obtain additional mechanistic insight into the contribution of tapasin structure to function, we solved complexes of tapasin bound to Fab fragments of two well-characterized anti-tapasin monoclonal antibodies, PaSta1^44^ and PaSta2^20^ (Table 1, Fig. 4). PaSta1 binds all tapasin molecules, whether free or bound to MHC-I^44^. By contrast, PaSta2 is unable to bind MHC-I-associated tapasin, indicating that it binds in the MHC-I binding site^20^. Consistent with prior immunoprecipitation data, PaSta1 and PaSta2 clearly bind to distinct sites on tapasin when evaluated with purified proteins by SPR (Supplementary Fig. 4). Both antibodies bind tapasin with *K*_D_ of 1 to 2 nM (Supplementary Fig. 4c, d). PaSta1 binds tapasin atop the N–IgV domain (Fig. 4a). This surface remains accessible in the tapasin–ERp57 complex (Fig. 4d). By contrast, PaSta2 contacts tapasin in the palm of the glove where MHC-I binds (Fig. 4b–d), interacting with both the N–IgV and the IgC domains at the tapasin hinge that joins the N–IgV domain to IgC (Fig. 4b). Although PaSta1 interacts with tapasin largely through the CDR3 of its H chain, PaSta2 predominantly exploits its L chain CDR1 for the interaction (Supplementary Figs. 5, 6). Comparison of these two Fab-complexed structures with those of tapasin–ERp57 (PDB 3F8U) and the tapasin–B44:05 structure reported here provides an opportunity to examine domain movements and loop flexibility in tapasin.

### Comparison of multiple structural models reveals dynamic domains and loops

**Fig. 5:**
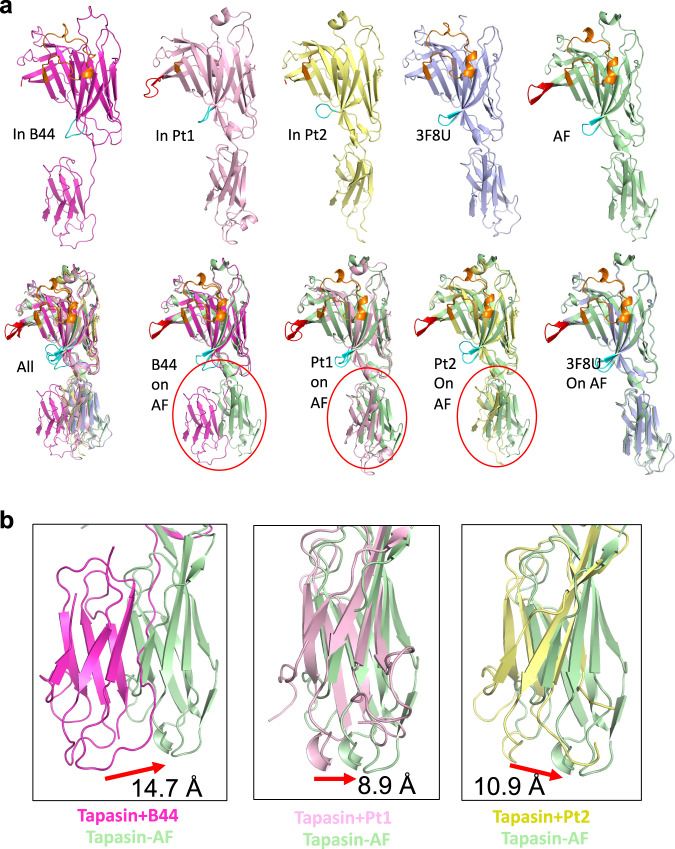
Comparison of Tapasin – Domain movements. **a** First row: ribbon illustration of tapasin from complexes: tapasin+B4405 (magenta)(7TUE), tapasin+PaSta1 (light pink)(Pt1, 7TUF), tapasin+PaSta2 (pale yellow)(Pt2, 7TUG), tapasin+Erp57 (3F8U, light blue), and AlphaFold model (from AlphaFold DB in March, 2022) of unliganded tapasin (AF, pale green); second row: all superposed based on N–IgV domains, and individual structures on AF. **b** IgC domain movement from AF: shift is 14.7 Å, 8.9 Å, and 10.9 Å for tapasin complexed to B4405, PaSta1, and PaSta2 respectively. (The AlphaFold model (AF-O15533-F1-model_v2.pdb) was based on the amino acid sequence of the compete protein (UniProt 015533), including signal peptide, lumenal domains, transmembrane region, and cytoplasmic domain. Residues 21-290, representing the N-IgV domain of the Tapasin model, were superposed on residues 1-270 representing the homologous structure of Tapasin in 3F8U (Tapasin-ERp57), 7TUE (Tapasin-B44:05), 7TUF (Tapasin-PaSta1), and 7TUG (Tapasin-PaSta2) in PyMOL.

We report here new structural models of tapasin based on X-ray diffraction data: tapasin–B44:05 and also two complexes with the antibodies PaSta1 and PaSta2. In addition to the experimentally determined model of tapasin–Erp57^20^, we also consider the computationally derived model of unliganded tapasin taken from the AlphaFold Database^45^. Comparison of these five structural models highlights the flexibility of the IgC relative to the N—IgV domain as tapasin interacts with different partners. The AlphaFold model, based largely on 3F8U, the tapasin–Erp57 heterodimer, serves as our unliganded standard. We compare the various models based on superposition of the N–IgV domains to visualize the potential for movement of the IgC domain, which is displaced by as much as 14.7 Å (for tapasin–B:44:05), 8.9 Å (for tapasin–PaSta1) and 10.9 Å (for tapasin–PaSta2)(Fig. 5b). These apparent translations are accompanied by IgC rotation of 16 to 18 degrees.

**Fig. 6:**
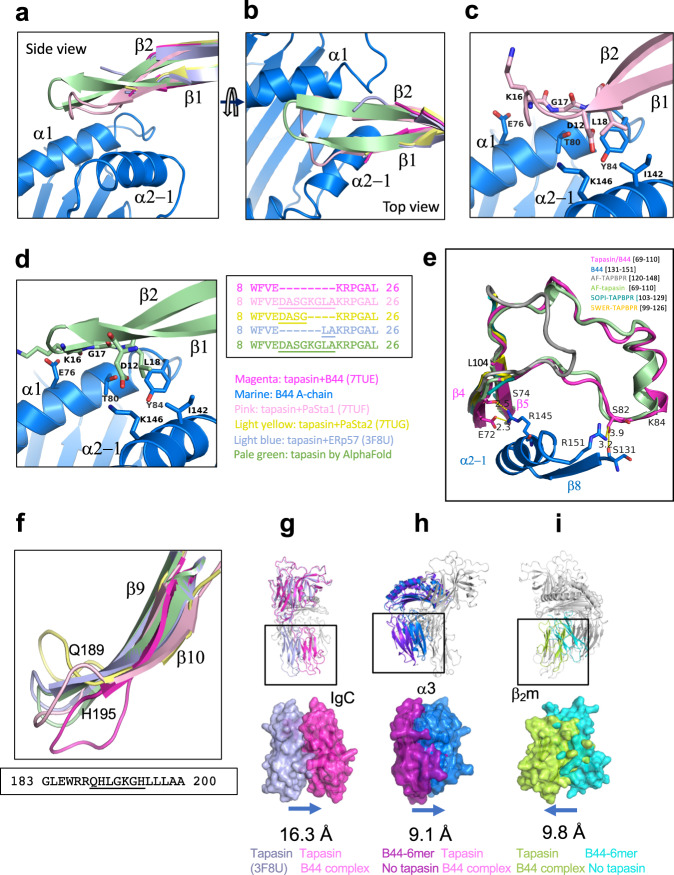
Loop movements and domain flexibility of tapasin. Four X-ray determined structures (3F8U, 7TUE, 7TUF, 7TUG) and the AlphaFold model (AF-015533) were superposed. **a**–**d** illustrate the **β**1 and **β**2 strands of tapasin (Trp8 to Leu26) lying atop the HLA-B44 **α**2-1 and **α**2 helices (**a**, **b**, **c**, and **d**)**. a** side view. **b** top view. **c** illustrates **β**1 and **β**2 (Trp8 to Leu26) from the tapasin–PaSta1(7TUF) complex. **d** AlphaFold model. As shown in the sequence alignment, only the tapasin structure from the PaSta1 complex (7TUF) and the AlphaFold computational model reveal a complete backbone through this region. The **β**1 and **β**2 strands clearly restrain (via Y84, T80, I142, and K146) the loop from descending into the B44:05 F pocket, and instead the loop provides a lid-like function. **e** shows the superposition of structures from Glu72 to Leu104 of tapasin for 7TUE, 5OPI (TAPBPR-MHC), 5WER (TAPBPR-MHC) and AF models of TAPBPR and tapasin. **f** superposition of **β**9 (Gly183-Arg188) and **β**10 (Leu196-Ala200) strands, and the hairpin loop (Gln189-His195) of the same structures. **g**, **h**, and **i** illustrate displacement of tapasin IgC, B44 α3, and β_2_m movement respectively. For comparisons shown in **h** and **i**, α1 res**i**dues 54-84 of unliganded B44:05 (7TUD) were superposed onto the same residues of tapasin–B44:05-T73C (7TUE) and displacement of α3 (**h**) and of β_2_m (**i**) was measured.

In addition, the multiple snapshots show that several loops vary among the different structures, revealing their potential for dynamic movement and adaptability when interacting with ligands. The first loop of interest, residues Glu11-Lys20 (Fig. 3c), is almost completely modeled in the complex with PaSta1 where one of the copies in the asymmetric unit shows continuous electron density (Supplementary Fig. 3c). This loop of tapasin is not in direct contact with the PaSta1 Fab. Superposition of tapasin from the PaSta1 complex on the tapasin–B44:05-T73C-6mer structure provides an illustration of the likely location of the Glu11-Lys20 loop when tapasin interacts with MHC-I. The Glu11-Lys20 loop of tapasin (Fig. 6a–d) is shorter than the Lys21-Arg36 loop of TAPBPR, remains suspended over the binding groove, and the central Leu18 is ~16 Å from the floor of the F pocket. These loop residues of tapasin interact mainly with α2−1 and α1 helices of B44:05 (Fig. 6a, c). Tyr84, Ile143, Lys146, Thr80 and Glu76 of B44:05 form a barrier that restrains the tapasin loop from direct access to the F-pocket (Fig. 6c). In the tapasin–B44:05 structure, the side chain of Tyr84 remains directed toward the F pocket of the peptide binding groove, and does not interact with tapasin, in contrast with the Tyr84-Glu102 interaction consistently seen in TAPBPR structures;^27,28^. These structural details are consistent with a peptide-trap mechanism^30^ and appear incompatible with the scoop-like mechanism originally proposed for the longer TAPBPR loop^27^ and extended to tapasin^23,37^. Examination of the AlphaFold model of tapasin, which is complete for the Glu11-Lys20 loop, leads to the same conclusion (Fig. 6d).

The second region for comparison is the strand/loop involving β strand 4, loop S74 to Leu104 and β5 of tapasin. As compared to TAPBPR, this loop is some 13 residues longer (Fig. 6e), and makes several critical interactions with residues of the MHC-I α2-1 helix, including H-bonds from tapasin Glu72 and Ser74 to B44:05 Arg145 and additional contacts between Ser82 and Arg151 of the MHC α2-1 helix, and Ser82 and Ser131 of the MHC-I β8 strand. The tapasin structure also reveals the contact of Glu72 with MHC-I Arg145. By contrast, the homologous Glu102 of TAPBPR contributes to the reorientation of MHC-I residue Tyr84.

The third region for comparison is the hairpin Glu189 to His195 that dives under the MHC-I β-sheet floor of the peptide binding groove and destabilizes the bound peptide (Fig. 3e). Superposition of this loop (189-195) from all available tapasin structural models (Fig. 6f) reveals considerable plasticity, depending on the particular molecule bound to tapasin.

The fourth major region that shows conformational flexibility and clearly influences the structural integrity of the peptide binding groove is where the tapasin IgC domain interacts with both the MHC-I α3 domain and the β_2_m subunit (Fig. 2f, Fig. 6g–i). This interaction generates a trimer of the three Ig-like domains due to the movement of the IgC domain of tapasin by 16.3 Å (Fig. 6f), of the α3 domain by 9.1 Å (Fig. 6h), and of the β_2_m subunit by 9.8 Å (Fig. 6i). This distortion of the β_2_m interaction with the MHC-I α chain contributes to the displacement of the floor of the peptide binding groove (Fig. 3d). A movie to show these domain movements was generated by morphing these structural models (Supplementary Movie 1).

To evaluate the distortion of the β_2_m–B44:05 α chain interaction due to tapasin in a solution environment, we recorded NMR TROSY spectra of selectively methyl-labelled β_2_m, in free form and complexed with the B44:05–6mer, as well as tapasin–B44:05-6mer (Supplementary Fig. 7). The NMR spectra reveal significant chemical shift differences (CSDs) in β_2_m upon assembly with the B44:05 α chain, as well as line broadening upon addition of tapasin to B44:05–β_2_m. The substantial CSDs of β_2_m residues Leu54 and Trp60 upon addition of tapasin are consistent with the lack of mobility of the 55 to 62 loop of β_2_m in the B44:05–β_2_m complex (Supplementary Fig. 7d). Line broadening of specific resonances on exposure of B44:05–β_2_m to tapasin likely indicates exchange between B44:05–β_2_m and a tapasin-bound form at an intermediate (microseconds to milliseconds) NMR timescale (Supplementary Fig. 7e). Consistent with our crystallographic analysis, we observed a significant broadening for the methyl resonance of Leu54 located beneath the floor of the MHC-I groove induced by interactions with tapasin, suggesting a plausible mechanism for weakening peptide binding affinity through perturbations of the β_2_m–B44:05 α2 domain interface.

## Discussion

This analysis of the interaction of tapasin with an MHC-I molecule adds considerable insight into the structural mechanisms that underlie peptide loading in the PLC. This extends our knowledge from multiple MD and structural studies of tapasin and the PLC^20–24,37^ as well as of TAPBPR^27–30,46–48^ to reveal a mechanism for tapasin-mediated peptide exchange which, although broadly comparable to that of TAPBPR, exploits structural features that are unique to tapasin. Several critical surfaces of tapasin engage MHC-I molecules both to stabilize peptide-receptive configurations and to destabilize peptide-bound ones. Tapasin exploits two major regions of the molecule for distinct purposes – the tapasin N–IgV unit grasps the peptide-binding domain of the MHC via the α2-1 helix and β-sheet floor, and the membrane proximal IgC domain repositions the MHC-I α3–β_2_m orientation. The N–IgV interaction may be visualized as a hand grasping the MHC-I platform domain with the index and middle fingers (connected by loop Glu11-Lys20) lying atop the α2-1 and α1 helices respectively (Supplementary Fig. 8a, d). Distinct from TAPBPR, this loop, visualized incompletely in the tapasin–MHC-I complex, but continuous in the tapasin–PaSta1 complex and in the AF tapasin model, is unable to approach the floor of the F pocket, thus favoring a trap model. The center of the palm (Supplementary Fig. 8b) (loop Glu72 to Lys84) engages the α2-1 helix, exploiting the strand/loop structure unique to tapasin, and is not found in TAPBPR. The thumb (loop Gln189-His195), exerting a function similar to that of the equivalent loop of TAPBPR, probes the MHC-I floor of β strands 6, 7, and 8 (Supplementary Fig. 8b). The heel of the palm (tapasin IgC domain) pushes the MHC-I α3 domain and β_2_m resulting in distortion similar to but quantitively far greater than that observed in the TAPBPR–MHC-I structures^27,28^ (Supplementary Fig. 8c). On engagement of high affinity peptide, tapasin reverts to an unliganded state, and MHC-I, now compact and stably loaded, is released (Supplementary Fig. 8e).

**Fig. 7:**
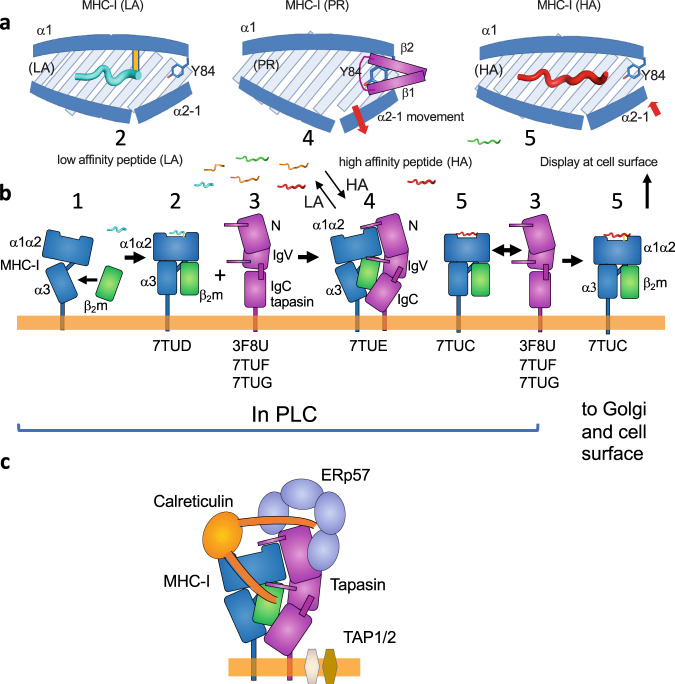
Structures represent distinct states of MHC-I and tapasin in the peptide loading pathway. **a** cartoons representative of (left to right) MHC-I bound to low affinity (LA) peptide, MHC-I in complex with tapasin (peptide receptive (PR) state), and MHC-I bound to high affinity (HA) peptide. **b** examples of distinct states of MHC-I–β_2_m and tapasin: **1**, MHC-I immediately after biosynthesis, prior to engaging any peptide (represented by molecules immunoprecipitated by 64-3-7^49^, which identifies the unfolded conformation of the α1 domain 3,10 helix^50^); **2**, MHC-I in conformed state bound to low affinity peptide (represented by our structure of HLA-B44:05-6mer (7TUD); **3**, tapasin in state not yet engaged with MHC-I (represented by tapasin complexed with ERp57 (3F8U), with PaSta1 (7TUF), or with PaSta2 (7TUG)); **4**, the MHC-I–tapasin complex (represented by 7TUE); **5**, MHC-I bound to high affinity peptide, released from tapasin complex (represented by 7TUC). **c** cartoon description of the tapasin–MHC-I complex as part of the PLC, based on^2^.

The X-ray structures we describe here address several molecular states of an MHC-I molecule (B44:05) and of its associated chaperone (tapasin) in the course of PLC stabilization and peptide acquisition in the ER (Fig. 7). Immunoprecipitation studies indicated early association with β_2_m and calnexin and exposure of a unique epitope of the α1 domain 3,10 helix prior to integration into the full PLC^49,50^ (Fig. 7b, state 1). The structure of the B44:05-6mer (Fig. 7b, state 2), (PDB 7TUD), represents one likely substrate for tapasin binding. A tapasin conformation prior to MHC-I engagement (Fig. 7b, state 3) is observed in tapasin–ERp57 (PDB 3F8U) and our two Fab complex strucures (PDB 7TUF, 7TUG). The X-ray structure of the complex of tapasin–B44:05 captures a putative metastable, peptide receptive B44:05 conformation (Fig. 7b, state 4), (PDB 7TUE). This conformation in the tapasin–B44:05 complex is remarkable for the structural differences from the B44:05-6mer structure noted above–namely, the lack of detectable bound peptide, the distortion of the F pocket region, the movements of the α2-1 helix and distortion of the binding groove, and reorientation of β_2_m. On binding a high affinity peptide (Fig. 7b, state 5)(7TUC), the mature conformation of the pMHC-I complex is released. These structural snapshots support a refined model of the steps that contribute to peptide acquisition by MHC-I molecules in the PLC, and may contribute to an understanding of the variable tapasin dependencies of HLA allelomorphs^19,32^, informing our general understanding of antigen presentation.

## Methods

### Plasmids

DNA encoding human tapasin (UniProtKB–O15533) encompassing the signal peptide, residues Gly1 to Glu381 of the lumenal domain (numbered as in^20^), a 3 C cleavage site, a BirA biotinylation sequence, a 6X HIS tag, and a termination codon was inserted between EcoRI and ApaI sites of pMT/V5-His A vector (ThermoFisher Invitrogen, Carlsbad, CA, USA). A bacterial expression plasmid encoding HLA-B*44:05 was kindly provided by Dr. John Altman from the collection of the NIAID/NIH Tetramer Core Facility. A stop codon was inserted following Trp274 to prevent inclusion of the BirA biotinylation sequence in the translated protein. Additionally, the codon encoding Thr73 was replaced with a cysteine codon to yield a T73C mutant for refolding with cysteine-containing truncated peptides. Mutagenesis was performed with the QuikChange Lightning Multisite mutagenesis kit (Agilent, Santa Clara, CA, USA). Plasmid encoding human β_2_m was described previously^26^.

### Protein expression and purification

The tapasin expression construct was transfected into *Drosophila* Schneider 2 (S2) cells grown in Insect X-Press medium (Lonza, Walkersville, MD, USA) together with plasmids encoding puromycin and blasticidin resistance. Cells surviving selection on culture medium containing puromycin and blasticidin were expanded, and protein expression was induced with 1 mM CuSO_4_ for three to four days. Tapasin was purified from the culture media by affinity chromatography on a chelate agarose column (ABT, Madrid, Spain) followed by size exclusion chromatography (SEC) on Superdex 200 (Cytiva, Uppsala, Sweden) in TRIS buffered saline (10 mM TRIS pH 8, 150 mM NaCl). SEC-purified tapasin was further purified by anion exchange chromatography on a Shodex QA-825 column developed with a gradient of 50 mM to 1 M NaCl in 25 mM TRIS pH 8. The BirA and 6X His sequences were removed by treatment with 3 C protease (Pierce, Rockford, IL, USA) overnight at 4 ^o^C prior to crystallization trials.

### MHC-I (hβ_2_m HLA-B*44:05-T73C constructs)

Bacterial expression and refolding of MHC-I was as described previously^51^. Parental HLA-B*44:05 was refolded with hβ_2_m and the peptide EEFGRAFSF, (DP α (46-54)). HLA-B*4405-T73C was refolded with hβ_2_m and the truncated peptide EEFGRC with the inclusion of 2 mM Gly-Leu dipeptide (Bachem, Switzerland) during refolding, dialysis, and purification to stabilize MHC-I molecules^36^. In addition, the parental HLA-B*44:05 was refolded with hβ_2_m and the full-length DP α peptide.

### Antibodies

The expressed heavy (H) and light (L) chains of the anti-tapasin antibody producing hybridomas PaSta1 and PaSta2^20,44,52^ were identified by RT-PCR amplification and sequencing of cDNA (Creative Biolabs, Shirley, NY, USA). Genes encoding antibody H and L chains were cloned into pcDNA3.1 for secreted expression. H and L chain-encoding plasmids were mixed together at a ratio of 1:2, and transfected into Expi293F cells following manufacturer’s instructions (ThermoFisher Gibco, USA). Six days later culture medium was harvested, and antibodies were affinity purified on Protein G followed by SEC on a Superdex200Inc column (Cytiva, Uppsala, Sweden). For Fab preparation, sequences for a 6X HIS tag and stop codon were inserted following the second cysteine in the hinge region of the H chain using the Q5 Site Directed Mutagenesis Kit (New England Biolabs, Ipswich, MA, USA). Following transfection of Expi293F cells, Fab was affinity purified 6 days later from culture supernatants on a TALON metal affinity resin (Takara, Mountain View, CA, USA) followed by SEC on a Superdex200Inc column.

### Surface plasmon resonance

All SPR experiments were performed at 25 ^o^C on a BiaCore T200 (Cytiva, Uppsala, Sweden) in 10 mM HEPES pH 7.4, 150 mM NaCl, 3 mM EDTA, and 0.05% Surfactant P20 at a flow rate of 30 µL/min. For analyses of MHC-I–tapasin interactions, tapasin, biotinylated at its C-terminal BirA sequence following manufacturer’s instructions (Avidity LLC, Aurora, CO, USA) was captured to a level of 200 resonance units (RU) onto a streptavidin chip (Cytiva, Uppsala, Sweden). Graded concentrations of B44:02 or B44:05 loaded with a 9mer peptide (EEFGRAFSF), B44:02-T73C or B44:05-T73C covalently loaded with the C-terminally truncated peptide EEFGRC were offered to the captured tapasin. A concentration series from 15.6 nM to 500 nM was used for B44:02–9mer and B:4405-T73C–6mer and from 1 µM to 16 µM for B44:05–9mer. Regeneration was accomplished with 5 M NaCl. Binding of MHC-I to tapasin was also analyzed by single cycle kinetics using biotinylated tapasin bound to a streptavidin sensor chip SA (Cytiva, Uppsala, Sweden). For analyzing the interaction of the PaSta antibodies with tapasin, Fab of PaSta1 or PaSta2 were covalently coupled onto CM5 chips (Cytiva, Uppsala, Sweden) via NHS/EDC coupling chemistry to approximately 200 RU. Graded concentrations of tapasin ranging from 0.06 µM to 4 µM were injected over the Fab surfaces at a flow rate of 30 μL/min for three minutes followed by a two-minute dissociation phase. For epitope mapping experiments, approximately 50 RU of tapasin was captured on a PaSta1 or PaSta2 Fab surface following which PaSta2 or PaSta1 Fab, respectively, were offered to the captured tapasin. Sensor surfaces were regenerated with 0.1 M glycine pH 1.5. For analyzing MHC-I binding to tapasin captured on PaSta1 or PaSta2 surfaces, graded concentrations of B44:05-T73C assembled with the truncated peptide EEFGRC were offered to the captured tapasin at a flow rate of 30 μL/min for three minutes followed by a two-minute dissociation phase. Binding of MHC-I to tapasin was also analyzed by single cycle kinetics using biotinylated tapasin bound to a streptavidin sensor chip SA (Cytiva, Uppsala, Sweden). Sensorgrams were globally fitted to a 1:1 binding model as implemented in Biacore T200 Evaluation Software v3.1 and plotted with GraphPad Prism.

### Crystallization and data collection and refinement

Crystallization conditions were identified by screening hanging drops at 18 °C. Crystals of tapasin–B44:05-T73-6mer were grown in 2.0 M AmSO_4_, 0.1 M Tris, pH 8.5; while crystals of B44:05-T73-6mer alone and B44:05-T73C-9mer alone were obtained under various PEG conditions but the best were 25% PEG1500, 0.1 M SPG (succinic acid, sodium phosphate monobasic monohydrate and glycine), pH 8.5; and 16% PEG 3350, 0.1 M Tris, pH 8.0, 0.2 M Ca acetate, respectively. The crystals of the Tapasin–PaSta1 complex were grown in 10% PEG 4000, 0.1 M Hepes, pH 7.5, and crystals of tapasin–PaSta2 complex were grown in 17% PEG 10000, 0.1 M Bis-Tris, pH 5.5, 0.1 M Am acetate. The crystals of PaSta2 alone were grown in 14% PEG 6000, 0.1 M Na cacodylate, pH 6.0. Crystals were cryoprotected in mother liquor containing 10% ethylene glycol, and flash frozen in liquid nitrogen. Diffraction data were collected (at wavelength 1.033 Å, in N_2_ stream at ~ 100 K) at Southeast Regional Collaborative Access Team (SER-CAT) beamline 22ID at the Advanced Photon Source, Argonne National Laboratory and processed with XDS^53^ to 3.1 Å, 1.45 Å and 1.25 Å for the tapasin–B44:05-T73C–6mer complex, B44:05-T73–6mer and B44:05-T73C–9mer, respectively. Diffraction data for complexes of tapasin–PaSta1 and tapasin–PaSta2, and for PaSta2 alone, were processed to 2.8 Å, 3.9 Å, and 2.3 Å respectively. The structures were solved by molecular replacement with Phaser^54^ using tapasin from PDB 3F8U as the search model. For the complex of tapasin and B44:05-T73C, the tapasin model was separated into two domains: N–IgV and IgC for molecular replacement search. For PaSta2, 2UYL with all CDR loops removed was used as the initial search model. For PaSta1, H chain (VH and CH1) of 6J15 and L chain (VL and Cκ) of 4ZSO were used as the initial search models, also lacking CDR loops. After molecular replacement solutions were found, the sequences of the model were replaced with those of PaSta1 and PaSta2, and all CDR loops were rebuilt manually. These initial models were subjected to several rounds of refinement with Phenix^55^ interspersed with manual building in Coot^56^. For low resolution refinements, as in the complexes of tapasin–B44:05-T73C–6mer (3.1 Å) and tapasin–PaSta2 (3.9 Å), “reference” higher resolution models of tapasin (3F8U) and PaSta2 alone (2.3 Å) were used as restraints in Phenix^55^. The data of tapasin–B44:05-T73C–6mer (3.1 Å) in P6_1_ revealed a pseudo twin (h,-h-k,-l) with a fraction of 0.307; the data of PaSta2 alone (2.3 Å) also was pseudo twinned (h,-k,-l) with a fraction of 0.460. For tapasin–PaSta2 (3.9 Å), *B*-factor sharpening of 100 Å^2^ was applied in order to highlight high resolution details, the average *B*-factor was reduced to 73.6 Å^2^. R_work_/R_free_ (%) values for final refined models of B44:05-T73C–9mer, B44:05-T73C–6mer and tapasin–B44:05-T73C–6mer are 18.0/19.5, 19.2/21.3, and 28.4/30.8 respectively; R_work_/R_free_ (%) values for final refined models of tapasin–PaSta1, tapasin–PaSta2, and PaSta2 alone are 23.4/27.8, 28.4/32.3, and 21.8/24.0 respectively. Data collection and refinement statistics are summarized in Table 1. Graphics figures were generated with PyMOL.

### NMR-spectroscopy

NMR samples of free hβ_2_m, hβ_2_m–B44:05-T73C–9mer, hβ_2_m–B44:05-T73–6mer for resonance assignments and chemical shift mapping experiments were prepared using the following isotope-selective labeling schemes: Ile methyl (Ile ^13^Cδ_1_ in an otherwise U-[^14^N, ^12^C, ^1^H] background], AILV methyl (Ala ^13^Cβ, Ile ^13^Cδ_1_, Leu ^13^Cδ_1_/^13^Cδ_2_, Val ^13^Cγ_1_/^13^Cγ_2_ in an otherwise U-[^15^N, ^12^C, ^2^H] background), and ILV* (Ile ^13^Cδ_1_; Leu ^13^Cδ_1_/^13^Cδ_2_ and Val ^13^Cγ_1_/^13^Cγ_2_ in an otherwise U-[^15^N, ^13^C, ^2^H] background, using established protocols and reagents^57,58^. In all samples, the B44:05 heavy chain and peptide were at natural isotopic abundance, and the light chain hβ_2_m was isotopically labeled, as described previously for the hβ_2_m/HLA-A*02:01 system^58^. Samples were in the concentration range of 150 to 300 μM prepared in a standard NMR buffer (50 mM NaCl, 20 mM sodium phosphate pH 7.2, 10% D_2_O). Backbone and methyl resonance assignments were derived using a series of TROSY-based 2D and 3D experiments recorded at a ^1^H field of 600, 700 MHz or 800 MHz at 25 °C, following a multi-pronged approach described previously for a similar system^59^. Final methyl assignments were verified using an automated software, MAUS^60^. For tapasin chemical shift mapping experiments, we used a 1:1 tapasin: hβ_2_m–B44:05-T73–6mer ratio prepared in the NMR buffer conditions. All NMR data were processed with NMRPipe and analyzed using NMRFAM-SPARKY^61,62^.

### Reporting summary

Further information on research design is available in the Nature Research Reporting Summary linked to this article.

## Supplementary information

Supplementary Information

Description of Additional Supplementary Files

Supplementary Movie 1

Reporting Summary

## Data Availability

X-ray structure factors and refined coordinates have been deposited in the Protein Data Bank (www.wwpdb.org) under accession numbers: 7TUC, 7TUD, 7TUE, 7TUF, 7TUG, and 7TUH for B44:05-T73C–9mer, B44:05-T73C–6mer, Tapasin–B44:05-T73C–6mer, Tapasin–PaSta1(Fab), Tapasin–PaSta2(Fab), and PaSta2 (Fab) respectively. NMR assignments for free hβ_2_m, hβ_2_m–B44:05-T73C–9mer, hβ_2_m–B44:05-T73–6mer, and tapasin with hβ_2_m–B44:05-T73C–6mer have been deposited into the Biological Magnetic Resonance Data Bank (http://www.bmrb.wisc.edu) under accession numbers 51097, 51098, 51099, and 51100. Expression vectors for recombinant proteins are available at request from the authors. Source data are provided with this paper.

## Source data

Source Data
