# Supplementary material for: Structural mechanism of tapasin-mediated MHC-I peptide loading in antigen presentation
Source: Nat Commun. 2022 Sep 17;13:5470. doi: 10.1038/s41467-022-33153-8 (PMC9482634; doi:10.1038/s41467-022-33153-8)
Supplement: Supplementary file 1 — Supplementary Information [file 41467_2022_33153_MOESM1_ESM.pdf]

Structural mechanism of tapasin-mediated MHC-I peptide loading in antigen presentation

Jiang et al.

Supplementary Figures

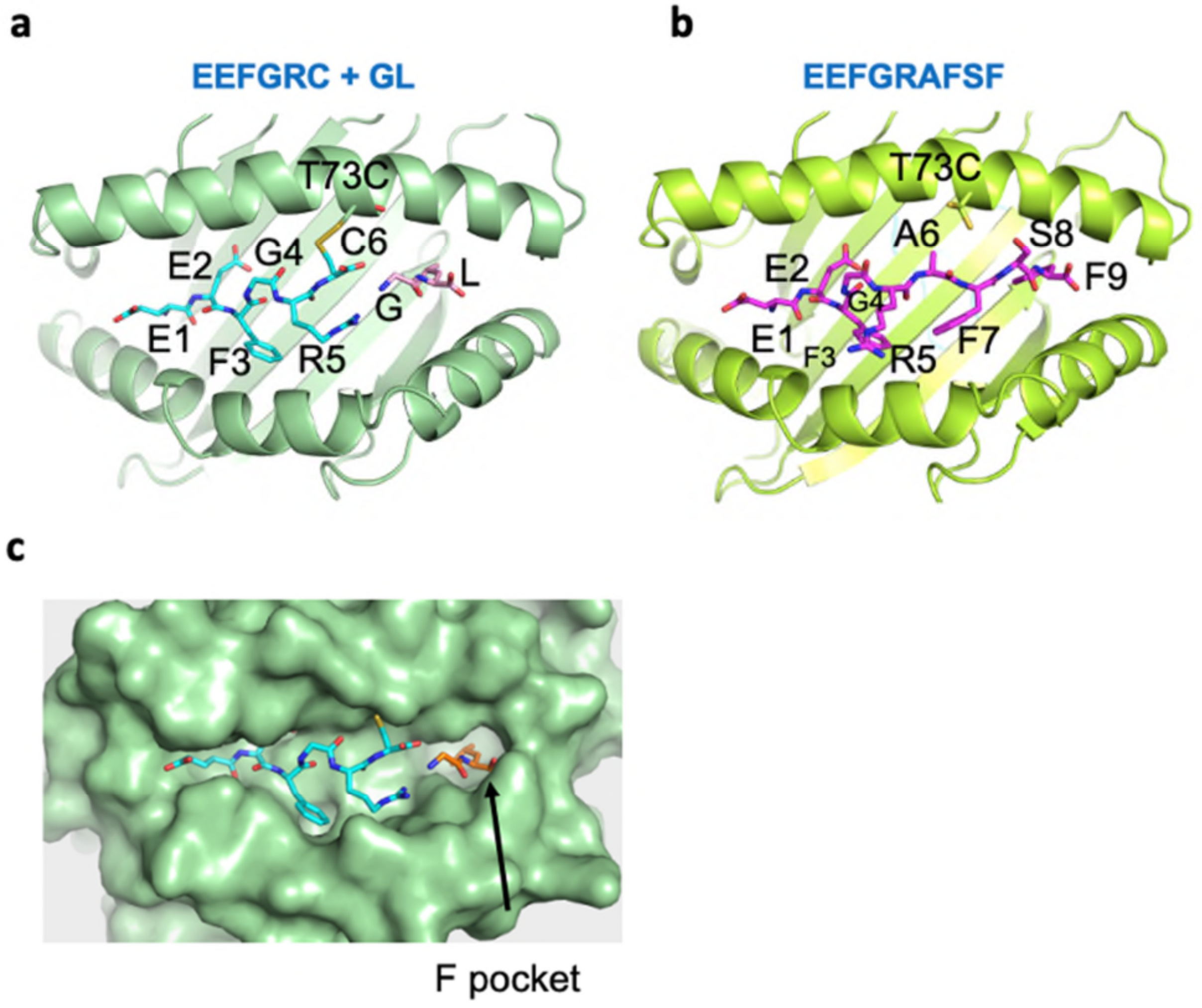

Supplementary Fig.1. Structures of unliganded B44:05-T73C–6mer and B44:05-T73C–9mer reveal peptides bound in canonical configuration. a graphics representation of B44:05-T73C–6mer (7TUD, 1.45 Å) and **b** B44:05-T73C–9mer (7TUC, 1.25 Å) bound to peptides EEFGRC and EEFGRAFSF respectively are shown, viewing the peptide binding groove in ribbon depiction and stick figure illustration of the peptides. The GL dipeptide is well-visualized in **a. c** illustrates location of the F pocket that accommodates the side chain of the Leu of the dipeptide, or the C-terminal Phe of the full-length peptide.

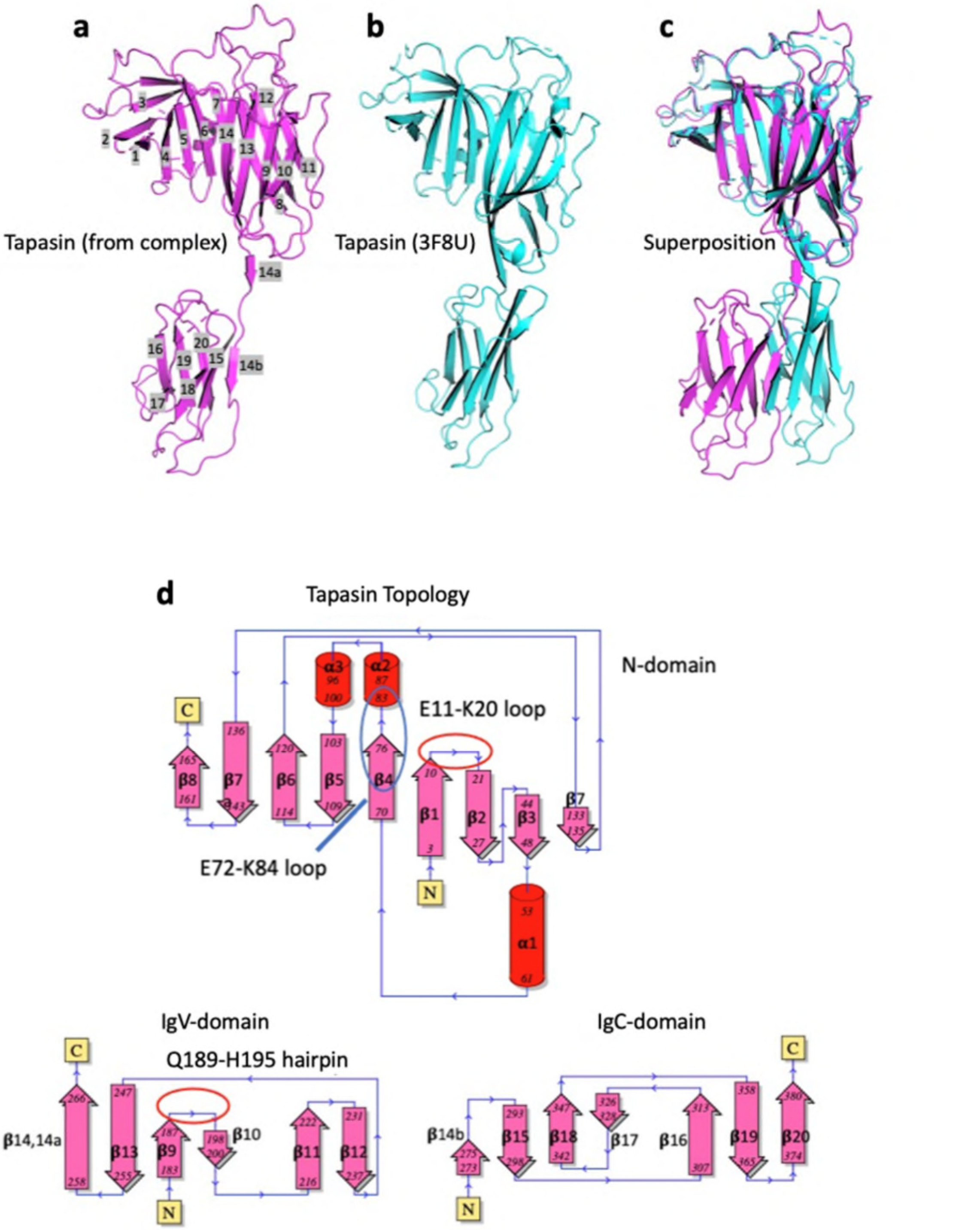

Supplementary Fig. 2. Secondary structure of Tapasin in complex with B44:05 compared to that of Tapasin bound to ERp57. a Ribbon display of Tapasin in B44:05 complex (7TUE). **b** Tapasin in ERp57 complex (3F8U). **c** and the superposition of the two, based on the N–IgV domain is shown a superimposition of 3F8U with Tapsin from B44+tapasin. **d** Topology of the three indicated domains, N, IgV, and IgC, is indicated as calculated by PDBSum[1]. (Note that N and IgV together form a clear superdomain). Strands are labelled consistent with Dong [2,] and loops E11-K20, E72-K84, and Q189-H195 are highlighted.

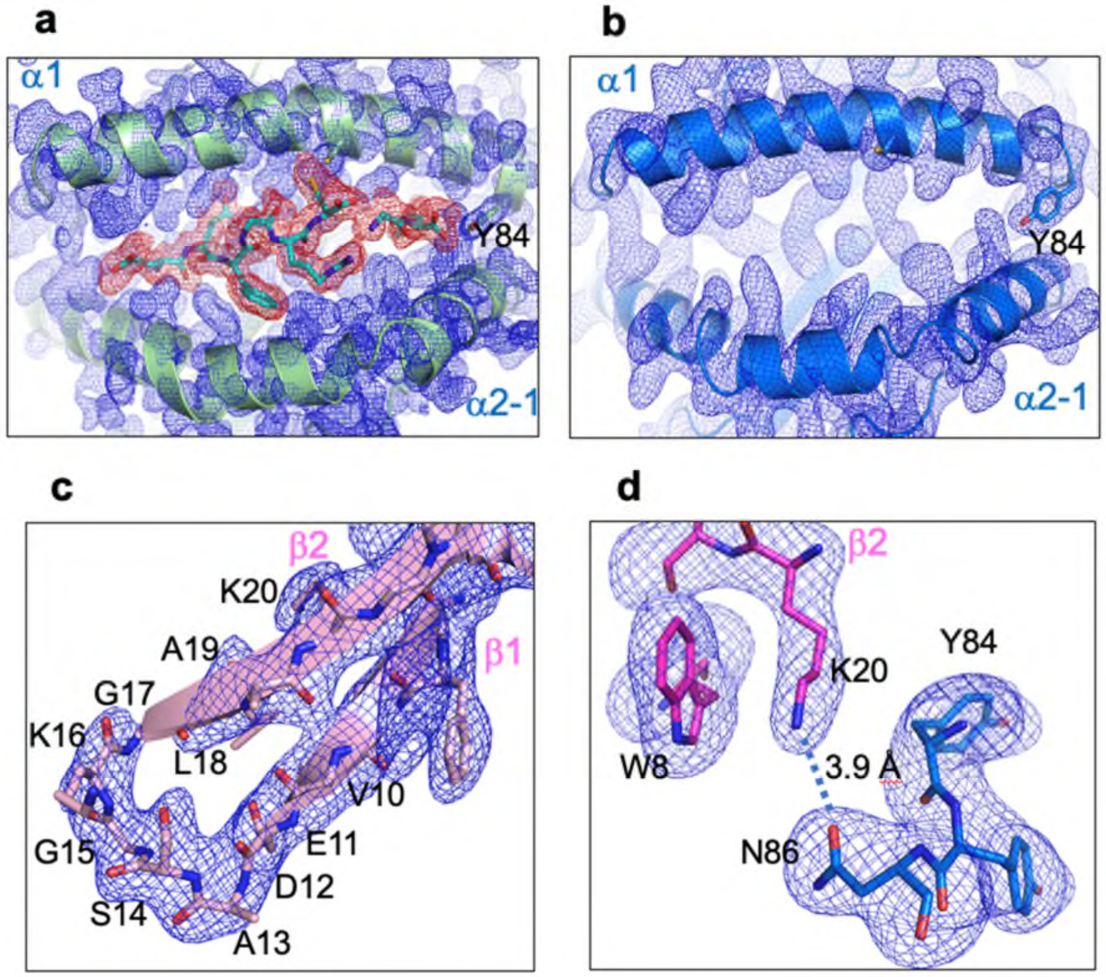

Supplementary Fig. 3. Electron density maps are vacant for bound peptide and Glu11-Lys20 loop in tapasin–B44:05 structure. Electrondensity maps (2mFo-DFc), were contoured at 1**σ**. **a** Peptide binding groove of Tapasin in complex with B44:05-T73C (7TUE). No electron density is observed for 6mer and dipeptide GL. Note good density for Tyr84. **b** Unliganded B44:05-T73C–6mer reveals clear density for 6mer peptide and GL dipeptide. **c** Full loop of Glu6-Ala24 of tapasin in complex with PaSta1 (7TUF). **d** Asn86 of B44:05 interacts with Lys20 of tapasin and forms a hydrogen bond.

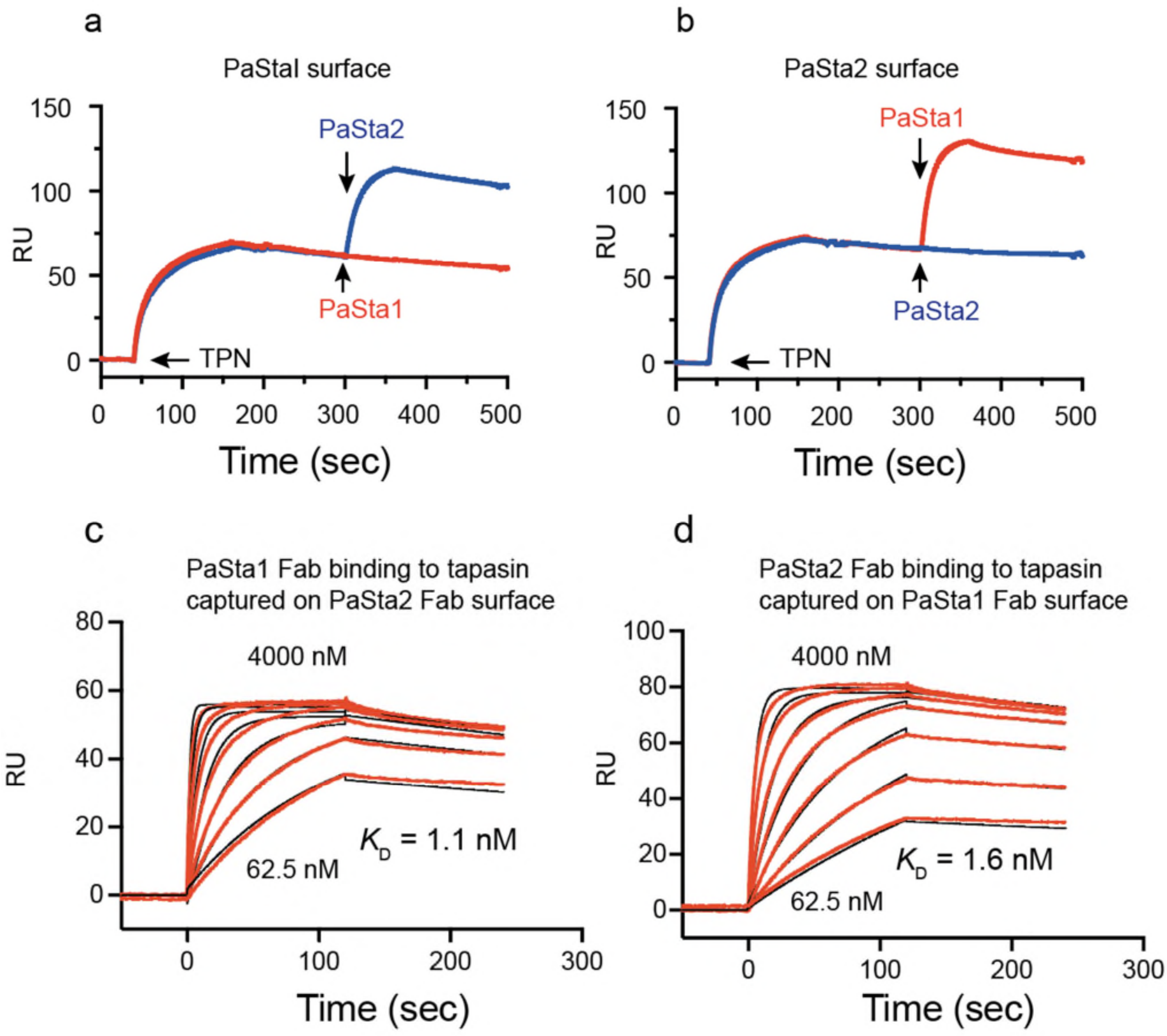

Supplementary Fig. 4. PaSta1 and PaSta2 bind to distinct epitopes on tapasin. a ~300 RU of PaSta1 Fab or **b** PaSta2 Fab were immobilized on a CM5 chip. Tapasin was then captured on both surfaces to a level of ~100 RU following which PaSta1 Fab (red) or PaSta2 Fab (blue) was injected over the captured tapasin. For analyzing the kinetics and binding affinities, the indicated concentrations of PaSta1 Fab **c** or PaSta2 Fab **d** were injected over tapasin captured by PaSta2 Fab or PaSta1 Fab respectively. Data are displayed in red and the fits to a 1:1 binding model as implemented in BiaEval 3.1 are overlaid in black. The experiments were repeated a minimum of two times and representative results are shown.

a

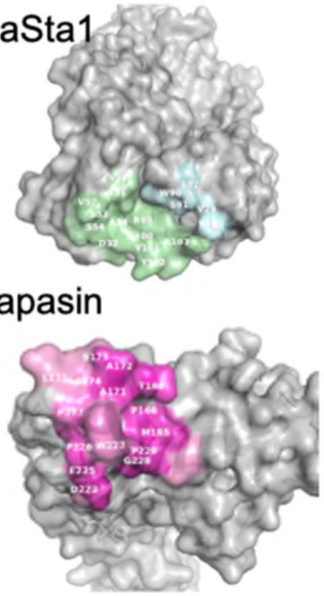

BSA (PaSta1) = 609 Å² (H) + 178 Å² (L) = 787 Å²

Contact Table

TPN	Pt1H	Dist
M165	Y104	3.48
P166	Y102	2.90
P167	Y102	3.72
T168	Y102	3.45
A171	Y101	3.61
A172	S53	2.41
S174	V57	3.61
L175	V57	3.38
D223	R103	3.02
E225	R103	3.44
W227	Y51	3.16
G228	R103	3.14
P229	R103	3.48
W230	R103	3.50
**TPN**	**Pt1L**	**Dist**
E225	S30	2.85
P226	S91	3.37
W227	W90	3.08

Pt1H	TPN	Dist
D32	T168	3.59
Y51	W227	3.16
S53	A172	2.41
S54	A172	3.48
V57	L175	3.38
R99	W227	3.16
Y101	A172	3.22
Y102	P166	2.90
R103	D223	3.02
Y104	M165	3.48
**Pt1L**	**TPN**	**Dist**
S30	E225	2.85
W90	W227	3.08
S91	E225	3.15
S92	P226	3.39

b

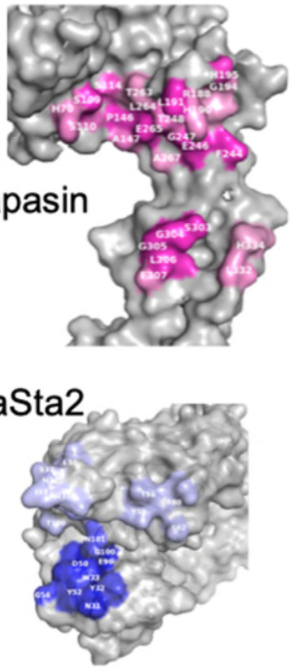

BSA (PaSta2) = 318 Å² (H) + 623 Å² (L) = 941 Å²

Contact Table

TPN	Pt2H	Dist
H190	G100	3.76
F244	Y32	3.24
E246	N101	2.90
**TPN**	**Pt2L**	**Dist**
H70	S32	3.55
S109	S32	3.59
S110	I33	3.37
P111	S32	3.69
S114	I33	3.68
P146	I33	3.48
H190	H97	3.69
L191	T100	3.68
T263	I33	3.35
E265	N34	3.70
S303	S62	2.68
G304	Y55	2.88
G305	T59	3.69
L306	R60	3.32

Pt2H	TPN	Dist
N31	F244	3.34
Y32	F244	3.24
G100	H190	3.76
N101	E246	2.90
**Pt2L**	**TPN**	**Dist**
S32	H70	3.55
I33	T263	3.35
N34	E265	3.70
Y55	G304	2.88
T59	G305	3.69
R60	G304	3.27
S62	S303	2.68
H97	H190	3.69
Y98	L191	3.72
T100	L191	3.68

Supplementary Fig. 5. Contact surfaces between PaSta1 and tapasin and PaSta2 and tapasin. a Surface of contacting residues of the interfaces of Tapasin with PaSta1 (7TUF), and **b** with PaSta2 (7TUG) (b) are shown. (For PaSta1 the first complex in the asymmetric unit is shown). Contacts to PaSta1 Heavy (Pt1H), PaSta1 Light (Pt1L), PaSta2 Heavy (Pt2H), and PaSta2 Light (Pt2L) are tabulated, based on evaluation of the indicated PDBs by CNS[3].

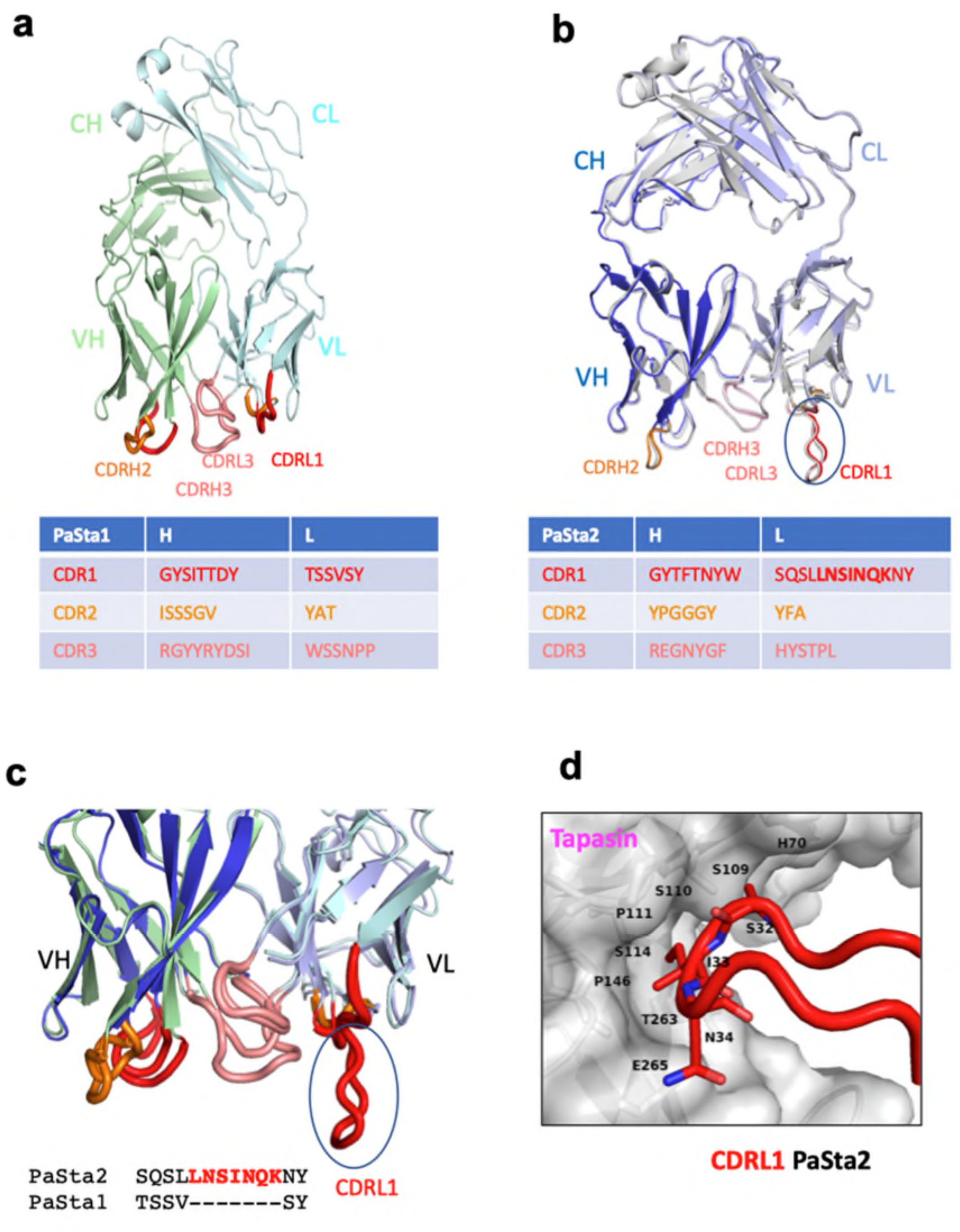

Supplementary Fig. 6. Comparison of tapasin–PaSta1 (7TUF), tapasin–PaSta2 (7TUG), and PaSta2 unliganded (7TUH) reveals differences in use of Fab H and L chains and conformational plasticity of CDR loops. a shows ribbon diagrams of Fab of PaSta1 from Tapasin–PaSta1 complex, indicating position and sequences of CDR loops. **b** Shows superposition of PaSta2 from liganded (Tapasin–PaSta2) and unliganded (PaSta2 alone, grey) structures, as well as CDR sequences. **c** shows superposition of liganded PaSta1 (green) and PaSta2 (blue) structures, emphasizing flexibility of CDR loops and lengthy CDRL1 of PaSta2. **d** shows interface of CDRL1 of PaSta2 with Tapasin.

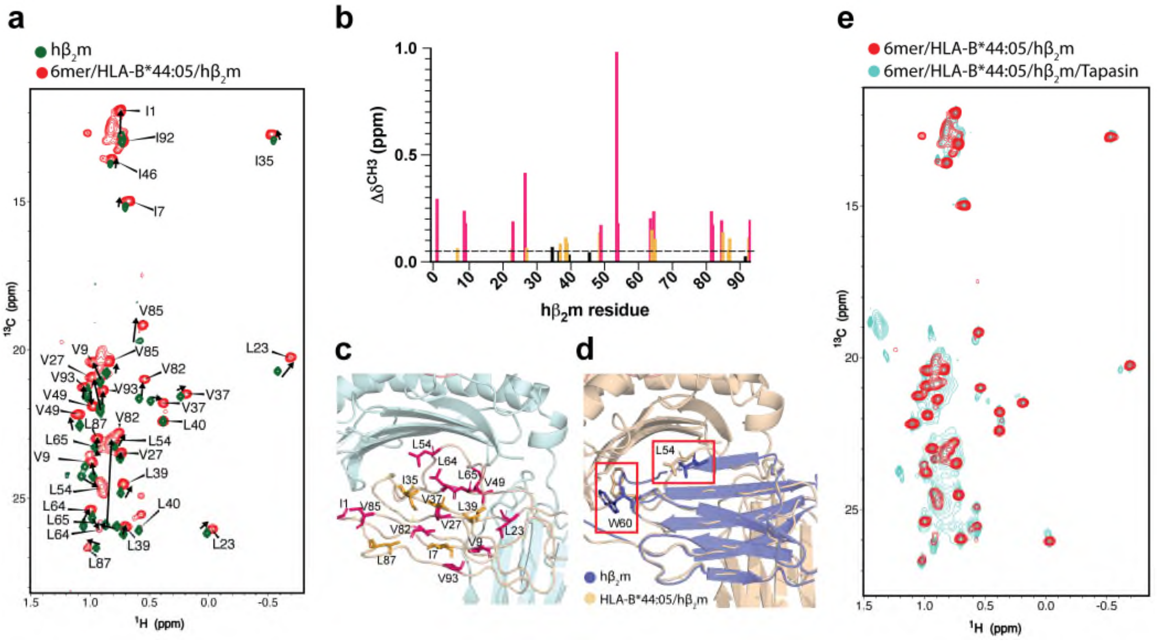

Supplementary Fig. 7. **Conformational changes in β_2m induced by interaction with B*44:05 and subsequent tapasin binding. a** 2D ^{1}H-^{13}C methyl TROSY spectra of $^{13}CH_3$-labelled human β_2m in the free (green) state and B*44:05-T73C–6mer (EEFGRC)-bound (red) states for ILV methyl probes. Both spectra were collected at 800MHz ^{1}H magnetic field and 25 °C. **b** Chemical shift difference (CSD, $\Delta\delta^{CH3}$, ppm) of human β_2m ILV methyls between free and B*44:05-6mer-bound states. Color coded orange if CSD was observed (CSD > 0.05ppm), with the most substantial CSD colored pink (CSD >0.15ppm). **c** CSD effects are plotted on the B*44:05-T73C-6mer– β_2m crystal structure (7TUD), with CSD changes colored orange and the most substantial CSD colored in pink. **d** Overlay of free human β_2m (PDB ID 2D4F) and B*44:05-T73C-6mer–β_2m (PDB ID 7TUD) crystal structures. Red boxes highlight a secondary structure change at L54 upon B*44:05 binding and a notable conformational change at the nearby W60, which likely contribute to the strong CSD observed at L54. **e** 2D ^{1}H-^{13}C methyl TROSY spectra of $^{13}CH_3$-labelled human β_2m in the B*44:05-T73C-6mer bound (red) and B*44:05-T73C-6mer–tapasin-bound (blue) states for ILV and AILV methyl probes, respectively. Spectra were collected at 800 MHz ^{1}H magnetic field (B*44:05-T73C-6mer–β_2m) or 700 MHz ^{1}H magnetic field (B*44:05-T73C-6mer–β_2m–tapasin), both at 25 °C.

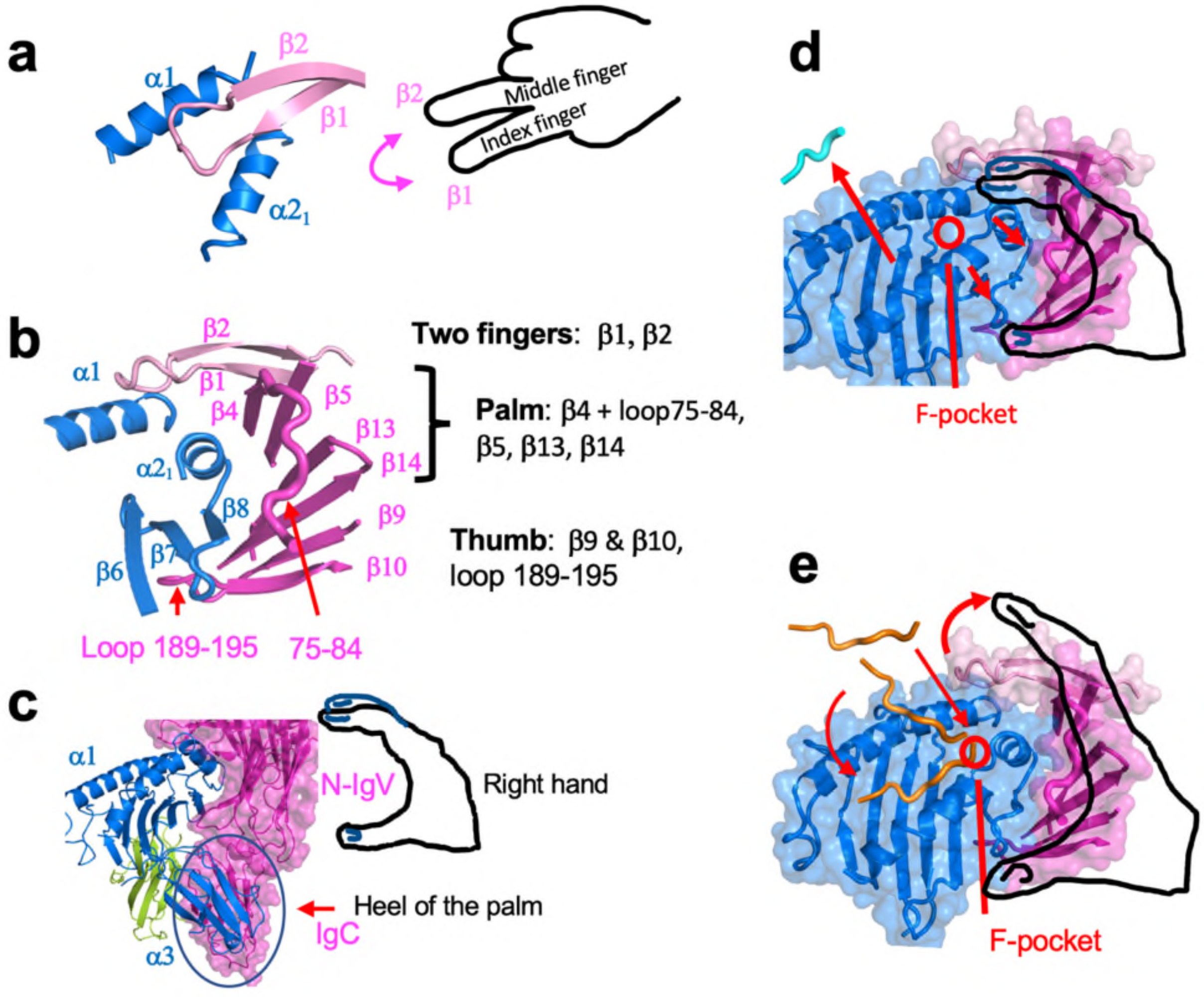

Supplementary Fig. 8. Illustration of mechanism of tapasin–MHC-I peptide loading by "Finger-Palm-Heel" interpretation. a β1, β2 strands atop the α2-1 and α1 helices are viewed as **t**wo fingers. **b** the palm consists of the β4 strand and loop 75-84, along with β5, β13 and β14; the thumb consists of β9, β10, and loop 189-195. **c** N-IgV domains are visualized as a right hand that grasps α2-1, part of α1 and the right-hand side of the peptide binding groove by inserting the thumb beneath the groove floor (strands β6, β7, and β6). **d** the interaction with α2-1 widens the groove facilitating the release of low affinity peptides. **e** on interaction of MHC-I with a high affinity peptide, α2-1 closes up around the peptide, the two fingers loosen their grip, and the MHC-I departs in the dissociation step.

B44-H chain	Tapasin	Distance (Å)
R111	K193	3.25
Y113	K193	3.23
N127	G194	3.31
E128	H195	3.50
S131	S82	3.16
A135	L250	3.31
A136	T263	3.17
Q141	Q261	3.25
I142	E11	3.12
I142	E72	3.33
Q144	Q261	3.13
R145	E72	2.32
R145	S74	2.49
P193	L294	3.19
P193	L296	3.24
S195	L294	3.39
H197	M277	3.49
T200	L296	2.98
R202	S341	3.04
E212	H190	3.34
T225	K270	3.44
Q226	K270	3.40
Q226	H299	3.46
E229	S298	2.89
E229	H299	3.44
E229	F300	3.16
E229	S339	2.46
E229	V340	3.37
E229	S341	3.02
V231	G338	3.30
V231	S339	3.21
R234	Y301	3.48
R234	P302	3.45
W244	G338	3.20
W244	S339	3.09
V248	V297	3.50
V248	S298	3.29

β_2m	Tapasin	Distance (Å)
K6	G338	3.21
I7	A331	3.22
Q8	A331	3.35
D96	W328	3.22
D96	L329	2.59
R97	W328	3.02
D98	W328	2.99
D98	H345	3.40
D98	Q347	3.21

Supplementary Table 1. Interchain contacts between Tapasin and B44:05 H chain and β_2m. Intermolecular contacts were calculated with CNS[3] using a 3.6 Å cutoff distance.

MHC-I Deleterious Mutants	Interpretation	Ref.
T143K	T143 not contact, but mutation confers steric clash	[4, 5]
Long strand-loop 128 to 136	Connects platform to a2-1 helix; N127, E128, S131, A135, A136 extensively contact tapasin long-strand loop and concave surface	[6]
Tapasin Deleterious Mutants		
TN3 (E72K0	Contact to I142, R145, in a2-1 helix	[2]
TN4 (E11K, D12R)	Not visualized in complex; loop hovers over a1 and a2 helices blocking sterically	[2]
TN5 (L250K)	Contacts to A135 on backbone, K expected to clash	[2]
TN6 (E185K, R187E, Q189S, Q261S)	Effect likely due to 261 as others are not contacts; Q261 contacts Q141 and Q142	[2]
L191R	Mouse polymorphism- doesn't bind PaSta2	[2]

Supplementary Table 2. Published mutants of MHC-I and tapasin, consistent with X-ray structure of tapasin-B44:05-T73C-6mer complex (7TUE).

SUPPLEMENTARY REFERENCES

1. Laskowski, R.A., Jablonska, J., Pravda, L., Varekova, R.S. & Thornton, J.M. PDBsum: Structural summaries of PDB entries. *Protein Sci* 27, 129-134 (2018).
2. Dong G, Wearsch PA, Peaper DR, Cresswell P, Reinisch KM: Insights into MHC class I peptide loading from the structure of the tapasin-ERp57 thiol oxidoreductase heterodimer. *Immunity* 2009, 30:21-32.
3. Brunger, A.T. *et al*. Crystallography & NMR system: A new software suite for macromolecular structure determination. *Acta Crystallogr D Biol Crystallogr* 54, 905-921 (1998).
4. Peace-Brewer AL, Tussey LG, Matsui M, Li G, Quinn DG, Frelinger JA: A point mutation in HLA-A*0201 results in failure to bind the TAP complex and to present virus-derived peptides to CTL. *Immunity* 1996, 4:505-514.
5. Lewis JW, Neisig A, Neefjes J, Elliott T: Point mutations in the alpha 2 domain of HLA-A2.1 define a functionally relevant interaction with TAP. *Curr Biol* 1996, 6:873-883.
6. Yu YY, Turnquist HR, Myers NB, Balendiran GK, Hansen TH, Solheim JC: An extensive region of an MHC class I alpha 2 domain loop influences interaction with the assembly complex. *J Immunol* 1999, 163:4427-4433.
7. Pettersen EF, Goddard TD, Huang CC, Couch GS, Greenblatt DM, Meng EC, Ferrin TE: UCSF Chimera--a visualization system for exploratory research and analysis. *J Comput Chem* 2004, 25:1605-1612.
