# Supplementary material for: Structural mechanism of tapasin-mediated MHC-I peptide loading in antigen presentation
Source: Nat Commun. 2022 Sep 17;13:5470. doi: 10.1038/s41467-022-33153-8 (PMC9482634; doi:10.1038/s41467-022-33153-8)
Supplement: Supplementary file 2 — Description of Additional Supplementary Files [file 41467_2022_33153_MOESM2_ESM.pdf]

Description of Additional Supplementary files

File Name: Supplementary movie 1

Description: Morph model depicting transition of B44:05–β2m-6mer complex (7TUD) to tapasin–B44:05-β2m complex (7TUE).le
